# Symptomatic Primary (AL) Amyloidosis of the Stomach and Duodenum

**DOI:** 10.1155/2013/525439

**Published:** 2013-02-25

**Authors:** Reidar Fossmark, Espen Skarsvåg, Harald Aarset, Henrik Hjorth-Hansen, Helge L. Waldum

**Affiliations:** ^1^Department of Gastroenterology and Hepatology, St. Olavs Hospital, Prinsesse Kristinas Gate 1, 7006 Trondheim, Norway; ^2^Department of Cancer Research and Molecular Medicine, Norwegian University of Science and Technology, Prinsesse Kristinas Gate 1, 7489 Trondheim, Norway; ^3^Department of Pathology and Medical Genetics, St. Olavs Hospital, Erling Skjalgssons Gate 1, 7006 Trondheim, Norway; ^4^Department of Haematology, St. Olavs Hospital, Prinsesse Kristinas Gate 1, 7006 Trondheim, Norway

## Abstract

Primary (AL) amyloidosis of the gastrointestinal tract is relatively rare, and symptomatic amyloidosis of the stomach is even more seldom. We present the case of a patient who was referred to upper endoscopy because of weight loss, nausea, and vomiting. Large areas of intramucosal hemorrhages were seen, and biopsies resulted in profuse bleeding stopped with endoscopic clips. The biopsies showed amyloid depositions and further workup revealed that the patient also had cardiac and neuropathic involvements. The patient started treatment with dexamethasone, melphalan and bortezomib. After treatment was started the nausea and epigastric discomfort improved, and a reduction in the biochemical markers troponin T, NT-proBNP, and M-component was observed. Gastric amyloidosis is rarely seen at upper endoscopy in patients without a previously established diagnosis, but the unusual endoscopic findings and bleeding tendency after biopsy should be kept in mind by gastroenterologists.

## 1. Introduction

Gastrointestinal involvement in amyloidosis is seen in primary (AL) amyloidosis, secondary (AA) amyloidosis, and dialysis-related (*β*2-microglobulin) amyloidosis. 

Primary (AL) amyloidosis of the gastrointestinal tract is relatively rare, and only 8% of patients were reported to have amyloidosis in biopsies from the GI tract, whereas only 1% had symptomatic amyloidosis of the stomach in a series of 769 patients [1]. Amyloidosis involving the gastrointestinal tract may cause symptoms related to altered motility, gastrointestinal bleeding, or malabsorption. In the stomach, gastric amyloidosis may have an endoscopic appearance mimicking gastric neoplasia [2, 3], hematomas, erosions and ulcerations, or a nodular gastritis [4]. The diagnosis of gastrointestinal amyloidosis may be hard to suspect in patients without previously diagnosed inflammatory or plasma cell disease. 

## 2. Case

A 74-year-old woman was referred to upper gastrointestinal endoscopy due to weight loss of 10 kg in 6 months, epigastric discomfort, nausea, and episodes of vomiting. She had a previous history of a tachy-brady syndrome resulting in pacemaker implantation two years before and received metoprolol treatment. 

**Figure 1:**
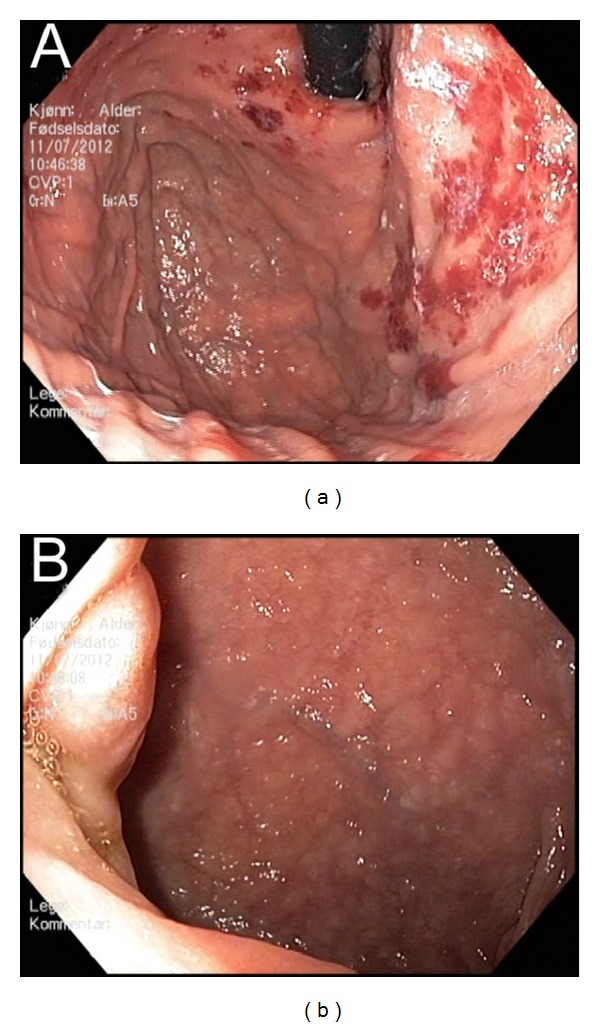
Endoscopic appearance of the corpus and cardia of the stomach with large areas of intramucosal hemorrhage (a) and of the duodenal bulb with a polypoid lesion (b).

**Figure 2:**
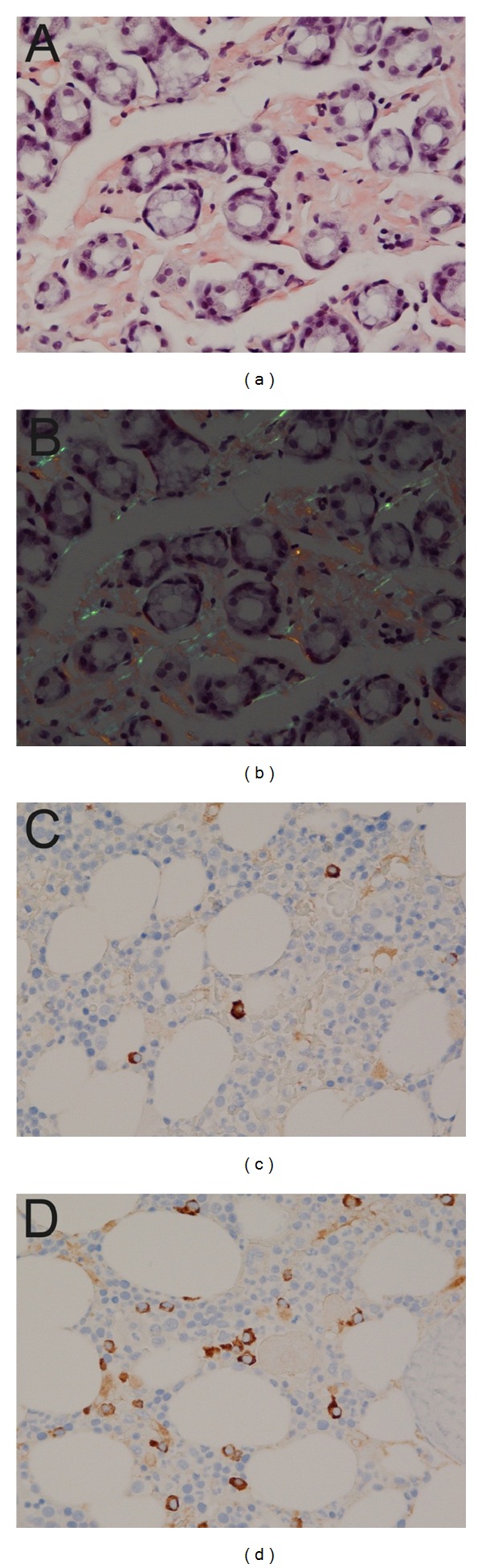
Biopsies from the gastric corpus stained with Congo red, with extracellular deposits between the gastric glands (a) and apple-green birefringence seen under polarized light (b). Immunohistochemical examination of a bone marrow biopsy showed a normal density of immunoglobulin kappa (*κ*) chain (c) and an increased density of immunoglobulin lambda (*λ*) chain positive cells (d).

Upper endoscopy showed large areas of intramucosal hemorrhage, mainly in the corpus and cardia of the stomach, whereas in the duodenal bulb, there was a polypoid lesion (Figure 1). A biopsy was taken from a small area with modest signs of intramucosal hemorrhage resulted in a profuse bleeding that was stopped with endoscopic clips. Biopsy collection from the polypoid lesion in the duodenum was apparently uncomplicated. In the evening, after the endoscopy, the patient had one episode of red hematemesis, but endoscopy the next day did not reveal the bleeding site, and Hb was 13.8 g/dL. Histological examination of the biopsies from the stomach showed amyloid deposits, and the lesion in the duodenal bulb was ectopic gastric mucosa with amyloid deposits (Figure 2). 

Subsequent diagnostic examinations revealed monoclonal component at serum electrophoresis quantified as immunoglobulin G (IgG) *λ* 6.4 g/L. Bone marrow biopsy showed a slight increase in plasma cells positive for light chain *λ* as a sign of monoclonal plasma cell expansion (Figure 2). These bone marrow changes did not fulfill criteria for multiple myeloma and were considered compatible with monoclonal gammopathy of unknown significance (MGUS). Further workup demonstrated considerable myocardial thickening by echocardiography with a reduced short axis contraction compatible with amyloid deposition. High troponin T (60 ng/L) and NT-proBNP (15000 ng/L) values were found, indicating stage III cardiac involvement, which has a dismal prognosis. Additionally, the patient had neuropathic pain, neurographic signs of axonal, and demyelinating sensorimotor polyneuropathy, also assumed to be caused by amyloid deposition. The diagnosis was hence AL amyloidosis with gastroduodenal, cardiac, and neuropathic involvements. The patient started treatment with dexamethasone, melphalan, and bortezomib which has been shown to induce a very high rate of deep biochemical response in multiple myeloma with improved survival [5]. Amyloidosis has previously been considered as a condition not amenable to treatment, but it is important to know that combinations of either melphalan or bortezomib with dexamethasone can induce organ responses in about 30–50% of patients with amyloidosis [6]. After treatment started, the nausea and epigastric discomfort improved and a reduction in biochemical markers such as troponin T, NT-proBNP, and M-component was observed. 

## 3. Discussion

Symptomatic gastric involvement is rare in patients with AL amyloidosis. This patient had large intramucosal hemorrhages and a polypoid lesion in the duodenum, where biopsies at upper endoscopy resulted in bleeding, but revealed amyloid deposits and led to the diagnosis of AL amyloidosis. Large hemorrhages requiring blood transfusion after biopsies from gastric amyloid lesions have been described by others [4] and may be related to small-vessel fragility due to amyloid infiltration and impaired hemostasis caused by factor X deficiency [7]. Bleeding diathesis has been observed in AL amyloidosis also under other circumstances and may cause purpura [8] and increase the risk of bleeding after liver biopsy [9]. 

The diagnosis of gastric AL amyloidosis should be considered in patients with plasma-cell disease. Gastric amyloidosis is rarely seen at upper endoscopy in patients without a previously established diagnosis, and only few endoscopic findings have been published, but the differential diagnosis should be kept in mind by gastroenterologists.
